# SAFE-MIL: a statistically interpretable framework for screening potential targeted therapy patients based on risk estimation

**DOI:** 10.3389/fgene.2024.1381851

**Published:** 2024-08-15

**Authors:** Yanfang Guan, Zhengfa Xue, Jiayin Wang, Xinghao Ai, Rongrong Chen, Xin Yi, Shun Lu, Yuqian Liu

**Affiliations:** ^1^ School of Computer Science and Technology, Xi’an Jiaotong University, Xi’an, China; ^2^ Shaanxi Engineering Research Center of Medical and Health Big Data, Xi’an Jiaotong University, Xi’an, China; ^3^ Geneplus Beijing Institute, Beijing, China; ^4^ Shanghai Chest Hospital, Shanghai Jiao Tong University School of Medicine, Shanghai, China

**Keywords:** EGFR, non-small cell lung cancer, target therapy, risk estimation, Hosmer-Lemeshow test, multi-instance learning

## Abstract

Patients with the target gene mutation frequently derive significant clinical benefits from target therapy. However, differences in the abundance level of mutations among patients resulted in varying survival benefits, even among patients with the same target gene mutations. Currently, there is a lack of rational and interpretable models to assess the risk of treatment failure**.** In this study, we investigated the underlying coupled factors contributing to variations in medication sensitivity and established a statistically interpretable framework, named SAFE-MIL, for risk estimation. We first constructed an effectiveness label for each patient from the perspective of exploring the optimal grouping of patients’ positive judgment values and sampled patients into 600 and 1,000 groups, respectively, based on multi-instance learning (MIL). A novel and interpretable loss function was further designed based on the Hosmer-Lemeshow test for this framework. By integrating multi-instance learning with the Hosmer-Lemeshow test, SAFE-MIL is capable of accurately estimating the risk of drug treatment failure across diverse patient cohorts and providing the optimal threshold for assessing the risk stratification simultaneously. We conducted a comprehensive case study involving 457 non-small cell lung cancer patients with EGFR mutations treated with EGFR tyrosine kinase inhibitors. Results demonstrate that SAFE-MIL outperforms traditional regression methods with higher accuracy and can accurately assess patients’ risk stratification. This underscores its ability to accurately capture inter-patient variability in risk while providing statistical interpretability. SAFE-MIL is able to effectively guide clinical decision-making regarding the use of drugs in targeted therapy and provides an interpretable computational framework for other patient stratification problems. The SAFE-MIL framework has proven its effectiveness in capturing inter-patient variability in risk and providing statistical interpretability. It outperforms traditional regression methods and can effectively guide clinical decision-making in the use of drugs for targeted therapy. SAFE-MIL offers a valuable interpretable computational framework that can be applied to other patient stratification problems, enhancing the precision of risk assessment in personalized medicine. The source code for SAFE-MIL is available for further exploration and application at https://github.com/Nevermore233/SAFE-MIL.

## 1 Introduction

Lung cancer holds the highest incidence rate among all types of cancer and boasts the largest selection of approved targeted therapeutic agents. Consequently, targeted therapy for lung cancer has become a prevalent and routine treatment in clinical practice. However, patients with target mutations show heterogeneous and diminished responses to the treatment (Cheng et al., 2020). Several studies have shown that its effectiveness is related to the gene mutation abundance level (Blakely et al., 2017; Samstein et al., 2019). Higher mutation abundance level may be related to better treatment outcomes (Zhou et al., 2011; Yan et al., 2019; Wang et al., 2021; Liu et al., 2022), such as a longer median survival time (Liu et al., 2022). In the event of a low abundance level, the therapeutic effect of targeted drugs may be reduced or completely lost (Tang et al., 2021). However, this issue remains unresolved in clinical practice. It is due to the absence of statistically interpretable tools that can accurately assess the risk of treatment failure in patients receiving targeted therapy. Furthermore, the optimal stratification threshold for targeted therapy based on mutation abundance level is unknown among the patient cohort. Hence, there is an urgent and unmet clinical need to develop a broadly applicable and statistically interpretable method approach capable of effectively assessing the risk associated with medicine utilization and then give the optimal threshold to assist clinical decision-making in target therapy.

Performing drug screening and selecting appropriate personalized treatment based on individual genomic, proteomic, and clinical features is one of the paramount goals of precision medicine (Nemati et al., 2018; Zhang et al., 2020; Banerjee et al., 2021; Sotudian and Paschalidis, 2022; Diao et al., 2023; Chen et al., 2024). Various clinical trials generate high quality results on effectiveness comparison of different drugs for the treatment of the same disease which is crucial for drug development and clinical practice (Rubin and Gilliland, 2012; Zhang Y. et al., 2021). Clinically, patients harboring same target gene mutations have different medication risks due to differences in mutation abundance level and drug sensitivity (Robichaux et al., 2021; Wang et al., 2021). The use of inappropriate drug regimens in patients with high risk may lead to delays in the patient’s condition and additional medical costs. One of the results is that mild patients may suffer severe or even catastrophic lesions (Schnipper et al., 2015; Daoud et al., 2020). Therefore, it is very necessary to assess the risk of medication use and utilize it in the clinical.

Nevertheless, this problem is different from the conventional drug effectiveness prediction problem. With the rapid development of artificial intelligence, a multitude of computational approaches to drug effectiveness prediction have been developed (Göttlich et al., 2016; Chang et al., 2022; Peng et al., 2022; Łosińska et al., 2022; Yang et al., 2023). Most of these approaches focus on utilizing the powerful feature extraction abilities and learning capabilities of artificial intelligence models to predict the drug responses of patients. Various computational models were employed to obtain potential vector representations of drugs and diseases for drug effectiveness prediction. However, a deep learning model is a black box, posing challenges in elucidating the acquired knowledge and the underlying principles guiding its predictive capabilities (Kuenzi et al., 2020). The existing drug effectiveness prediction models exhibit a tendency towards excessive complexity and lack relevant statistical interpretations, such as deep neural networks. According to Food and Drug Administration (FDA) policy, the assessment needs to be conducted in a manner that allows for statistical interpretation. Hence, this is a risk likelihood estimation problem rather than a prediction problem. Furthermore, the patient stratification problem is a matter of interest for both the clinical and drug regulatory communities, rather than the drug effectiveness prediction. Therefore, these existing drug effectiveness prediction models have high predictive accuracy; yet, their practical applicability in clinic practice is limited. Exploring the challenge of designing a reasonable model from the perspective of optimal patient stratification to consider the risk of treatment failure holds significant academic value.

The widespread application of multiple instance learning (MIL) in drug-target, drug activity, and drug effectiveness prediction (Fu et al., 2012; Zhao et al., 2013; Saberian et al., 2022; Wang et al., 2022) has attracted our attention. In clinical practice, the risk of drug failure is typically assessed on a population-wide scale, making it challenging to provide a personalized estimate of treatment failure risk for an individual patient prior to the initiation of medication. In MIL, the dataset is divided into groups of multiple instances, with the group labels known but the instance labels unknown, which has a good correlation with patient grouping in clinical drug practice. For example, suppose there are many microscopic tissue slice images used to detect cancer. Each image may contain multiple small regions, which we refer to as “instances.” The whole image can be labeled as “cancer” or “no cancer,” but it may be uncertain whether each specific small region contains cancer cells. In this case, we can consider each image as a “group” containing multiple “instance regions.” The algorithm needs to predict the label of the “group” based on the known labels of the “instances.” In the context of drug effectiveness prediction, using MIL to consider and model drug effectiveness may be a feasible idea.

This paper presents a generic novel framework termed SAFE-MIL for screening potential targeted therapy patients based on risk estimation, using epidermal growth factor receptor (EGFR)-mutant non-small cell lung cancer (NSCLC) as an example. The proposed framework integrates multi-instance learning with the Hosmer-Lemeshow test (Hosmer and Lemesbow, 1980) to estimate the risk of drug use, which provides statistical interpretability and can address the limitations of traditional methods. Experimental results demonstrate that SAFE-MIL provides interpretability and higher accuracy, hence potentially facilitating the process of therapeutic drug selection for patients.

## 2 Patients and methods

In this paper, we present a spontaneous formulation of the drug risk estimation problem as an instance of multi-instance learning. We address the problem in three stages, first organizing the given clinical information and drug action information of patients into instances, then identifying the estimated scores of all instances in the same group, and finally combining all the estimated scores as the output. Prior to these stages, data preprocessing was performed.

### 2.1 Patients and samples collection

In this research, we acquired three sets of Non-Small Cell Lung Cancer (NSCLC) sequencing data, exclusively sourced from EGFR-mutated NSCLC patients. Inclusion criteria for patients in this study: Patients must carry EGFR mutations (EGFRm) and be clinically selected for anti-tumor treatment with a tyrosine kinase inhibitor (TKI) based on the detection results. Exclusion criteria is that patients who have not received EGFR-TKI treatment according to the detection results, or patients carrying mutations in other drug target sites. The first batch consists of data from 100 patients with treatment-naive NSCLC (stage III–IV), all of whom underwent first-line targeted therapy. This information was gathered from 14 medical centers spanning the period from 23 February 2017, to 31 December 2019. The trial has been officially registered with the identifier NCT03059641. The second and third batches comprises data from 237 patients and 120 patients (stage IV) with EGFR-mutated NSCLC who underwent next-generation sequencing at Geneplus-Beijing (Beijing, China) between 2016 and December 2019 (Table 1). Tumor tissue samples were utilized to identify actionable mutations for targeted therapy in these patients. Notably, all these patients received anti-EGFR targeted therapy. It’s important to note that all participants in the study provided written informed consent. The research was conducted in accordance with a protocol approved by the Institutional Review Board of Shanghai Chest Hospital. Cohort 1 is the first batch of NSCLC patients were collected. In order to better generate multi-instance datasets, we conducted experiments using cohort 1 as independent data.

**TABLE 1:** Demographic and baseline characteristics of cohort-1,2,3.

Participants (n = 457)						
	Cohort1(n = 100)		Cohort2 (n = 237)		Cohort3(n = 120)	
Gender	NO.	%	NO.	%	NO.	%
Female	64	64	142	60	68	57
Male	36	36	95	40	52	43
Age (26–86)						
>60	56	56	111	47	58	48
<=60	44	44	126	53	62	52
Stage						
III	10	10	0	0	0	0
IV	90	90	237	100	120	100

Cohort 3 comprised 120 patients with NSCLC accepted EGFR-TKI target therapy (43.3% male, stage IV) (Supplementary Table S5), with ages at diagnosis ranging from 35 to 83 years. In the tumor samples, a total of 952 SVs are included (Supplementary Table S6). All patients were found to carry EGFR-sensitive mutations. Cohorts 1 and 2, 3 have recorded slightly different survival information, with the former documenting patients’ progression-free survival (PFS), while the latter records time to treatment failure (TTF).

DNA extraction, targeted capture, and NGS Genetic analysis for all data were performed as previously described (Nong et al., 2018; Zhang et al., 2019). Sequencing libraries were prepared from genomic DNA were prepared using Illumina TruSeq DNA Library Preparation Kits (Illumina) or MGIEasy Universal Library Prep Set (MGI Tech). Libraries were hybridized to custom-designed biotinylated oligonucleotide probes (Integrated DNA Technologies, Inc) targeting 1,021 genes. Prepared libraries were sequenced on a NextSeq CN 500 (Illumina) or MGISEQ-2000 sequencer (MGI Tech, Shenzhen, China). After the entire run was completed, image analyses, error estimation and base calling were performed to generate primary data. We then removed a few unqualified sequences from the primary data using a local dynamic programming algorithm, which included low-quality reads, defined as reads that contained more than 10 percent Ns in the read length, 50% reads with a quality value of less than five and with an average quality of less than 10 and adapter sequences including indexed sequence. The remaining sequences were termed as clean reads for further analysis. The clean reads were aligned to the reference human genome (hg19) with Burrows-Wheeler Aligner (version 0.7.12-r1039). Variants were called with GATK (version 3.4–46-gbc02625) and MuTect (version 1.1.4). Contra (v2.0.8) was used to detect copy-number variants (CNVs), and NCsv (in-house software version 0.2.3) was used to detect structural variants (SVs). Targeted capture sequencing required a minimal mean effective depth of coverage of 300 in tumor tissues Variants were filtered to exclude synonymous variants, known germline variants in dbSNP, and variants that occur at a population abundance level of >1% in the Exome Sequencing Project.

### 2.2 The model and algorithm

The SAFE-MIL framework was used, which utilizes multiple instances learning to predict drug failure risk in patients. The main steps of the SAFE-MIL framework include:

i. Designing drug effectiveness labels based on clinical features of patients.
ii. Building a model using multiple instances learning to predict drug failure risk by aggregating the labels of each patient.
iii. Using a new loss function based on the Hosmer-lemeshow test to capture differences between groups and improve the performance and statistical interpretability of the model.
iv. Learn the relationship between mutation abundance level and drug failure risk, and determine the optimal positive threshold for drug failure.

Cohort 1 is the first batch of NSCLC patients we collected. In order to better generate multi-instance datasets, we conducted experiments using cohort 1 as independent data. We randomly divided cohort 1 into a training set and a testing set and conducted independent experiments. Similarly, we subsequently collected more samples (cohort 2 and cohort 3) and conducted similar independent experiments. Due to slight differences in collection time, clinical features, and other aspects among these three cohorts, we did not merge them.

### 2.3 The design of drug effectiveness labels for patients

In order to better illustrate the variations in therapeutic efficacy among different patients, we have considered representing the therapeutic efficacy of a drug as a probability value ranging from 0 to 1. Initially, we evaluated the correlation between patients’ clinical characteristics and the effectiveness of the drug based on existing literature. Given that a patient’s drug response is closely linked to their PFS (Paz-Ares et al., 2018), we utilized the patients’ PFS to determine the drug’s effectiveness for each individual of cohort 1. For cohort 2, we attempted to assess the efficacy of the drug using TTF. To begin, patients with a PFS/TTF exceeding 8 months as of the cut-off value were included in the experimental cohort. Subsequently, we employed a min-max scaling technique to map the PFS/TTF values of these patients onto a scale of 0–1. This scaled value was then used to represent the probability of the patient’s drug efficacy.

### 2.4 Data preprocessing and patient grouping

The data preprocessing process is shown in Figure 2A. Given that the efficacy of a medication for an individual patient remains unobservable prior to treatment, we propose an unsupervised clustering approach to categorize patients with analogous characteristics into distinct cohorts. Subsequently, we employ multi-instance learning to estimate the probability of drug effectiveness across these patient groups. We used the K-means unsupervised clustering algorithm to group patients into four clusters (details see Supplementary Materials S1, S2, Figure S1). We then randomly sampled 1–10 patients with replacement to form a group, with each patient serving as an instance. We created datasets based on the two aforementioned patient cohorts. For each cohort, we generated two datasets of varying sizes to demonstrate the model’s generalization capacity. One dataset comprised 600 bags (cohort 1–600 and cohort 2–600), with 150 bags randomly generated from each cluster. The other dataset included 1,000 bags (cohort 2–1,000 and cohort 2–1,000), with 250 bags randomly generated from each cluster. The drug effectiveness of the groups was determined by calculating the average value of the drug effectiveness label assigned to the instances in the group. The raw input data, preprocessed output data, and grouped data can be accessed at GitHub repository.

### 2.5 Objective function

Here, risk estimation is formulated as a regression problem. We combine multiple instances learning and Hosmer-lemeshow test to design our model. Suppose that the training set consists of *N* training groups {*G*_*1*_, *G*_*2*_, …, *G*_*N*_} and each group contains *M*_*i*_ instances {*G*_*i1*_, *G*_*i2*_, …, *G*_*iMi*_}, where the label corresponding to each training group *G*_*i*_ is marked *L*_*i*_. In addition, each instance in the groups is a *p*-dimensional attribute value vector.

In multi-instance learning, the task is to predict the labels of unseen groups. The learning algorithm can acquire the label of the group, but cannot obtain the label of the instance. Therefore, we define the global error function of the neural network at the group level using the labels of the training groups as

$$E=\sum_{i=1}^{N}E_{i}$$ (1)

Where *E*_*i*_ is the output error corresponding to the group *G*_*i*_ (Equation 1).

To enhance the statistical interpretability of our framework and establish trust between clinicians and our framework, we consider designing loss functions based on statistical tests. We found that the Hosmer-Lemeshow test (Hosmer and Lemesbow, 1980), which evaluates the goodness-of-fit of grouped data, is closely related to multiple instance learning. The Hosmer-Lemeshow test is widely used in the evaluation of risk models (Kramer and Zimmerman, 2007; Zhang L. et al., 2021; Davies et al., 2022). Given the excellent performance of the Hosmer-Lemeshow test in model verification, we design the loss function according to the calculation formula of Hosmer-Lemeshow (HL) statistic (*HL*_*s*_) (Equation 2). The Hosmer-Lemeshow test is a statistical method used to evaluate the goodness-of-fit for binary logistic regression models. The basic idea is to divide the data into several groups and then compare the actual observed values with the predicted values in each group. In the Hosmer-Lemeshow test, the *p*-value is calculated based on the chi-squared distribution, corresponding to the Hosmer-Lemeshow statistic’s cumulative distribution function value. If the *p*-value is very small (less than 0.05), it indicates that the model does not fit well, meaning there is a significant difference between the predicted probabilities and the actual observed values. If the *p*-value is large, it indicates that the model fits well, meaning there is no significant difference between the predicted probabilities and the actual observed values. Suppose that the observed value (*Observed.A*, *Observed.not.A*) and expected values (*Expected.A*, *Expected.not.A*) of *Event A* are divided into Q groups {*G*_*1*_, *G*_*2*_, … , *G*_*Q*_}, the HL statistic and *p*-value (*HL*_*p*_) of HL test are defined as

$${HL}_{s}=\sum_{q=1}^{Q}\left(\frac{{\left(Observed.A-Expected.A\right)}^{2}}{Expected.A}+\frac{{\left(Observed.not.A-Expected.not.A\right)}^{2}}{Expected.not.A}\right)$$ (2)

$${HL}_{p}=1-chisq\left({HL}_{s},Q-2\right)$$ (3)

where *chisq* (*HL*_*s*_, *Q*-2) is the chi-square distribution with *Q*-2 degrees of freedom (Equation 3 more details in Supplementary Material S3).

Since the HL based loss function (HL loss) is partially non-differentiable, we use the mean square error loss function to replace the non-differentiable points of the HL loss function (Equation 4). Research indicates that the output value of a group in neural network is determined by the maximum output value of the instance in the group (Amar et al., 2001). Therefore, to simulate the above rules, the output error of group *G*_*i*_ is defined as

$$E_{i}=\left\{\begin{array}{l} \frac{{\left(\max\left(o_{ij}\right)-L_{i}\right)}^{2}}{\max_{0\leq j\leq M_{i}}\left(o_{ij}\right)}\;\max_{0\leq j\leq M_{i}}\left(o_{ij}\right)\neq 0\\ \frac{1}{2}{\left(\max_{1\leq j\leq M_{i}}\left(o_{ij}\right)-L_{i}\right)}^{2}\;\max_{0\leq j\leq M_{i}}\left(o_{ij}\right)=0 \end{array}\right.$$ (4)

where *o*_*ij*_ is the network output corresponding to the instance *G*_*ij*_.

The input of the model is all the training groups, and the output is the drug effective probability of the patients in the group, which is used to measure the patient’s drug use risk (1- drug effective probability). The hyperparameters used for SAFE-MIL are summarized in Table 2. In addition, we explored the correlation between the risk stratification and the survival benefits of the patients. In all analyses, *P* < 0.05 was considered statistically significant.

**TABLE 2:** Hyperparameter of the SAFE-MIL.

Hyper-parameters	Setting
Learning rate	0.001
Epochs	200
Optimizer	Relu
Hidden layer neuron	50

In addition, after training the model, we search for the optimal positive threshold that can differentiate the drug use risk of patients based on the model’s outputs on the training set. Firstly, we define the search space for the optimal positive threshold as [ ${MA}_{margin}$ - *margin*, + ${MA}_{margin}$ *margin*], where MA represents the mutation abundance and the margin boundaries determine the size of the search space (Equation 5). The objective function of optimal positive threshold is defined as follows:

$$diff=\max_{t}\left|\frac{1}{j-t}\sum_{i=t}^{j}d_{i}-\frac{1}{t}\sum_{i=1}^{t}d_{i}\right|$$

$$\tau =T_{t}$$ (5)

Where $d_{i}$ represents the drug failure risk, and τ represents the optimal positive threshold. When t is set to $T_{t}$ , the samples are divided into two groups based on τ , and the average drug failure risk difference between them is the largest.

### 2.6 Baselines

We evaluated SAFE-MIL with the four classical regression loss functions, including mean absolute error loss (MAE loss) (Fisher, 1915) (Equation 6), mean square error loss (MSE loss) (Equation 7) (Fisher, 1921), Huber loss (Equation 8) (Huber, 1992) and Log-cosh loss (Equation 9) (Shen et al., 2018). During one neural network iteration, the formulas of the four regression loss functions are defined as

$$Mse\;loss=\frac{1}{2}{\left(y_{p}-y_{t}\right)}^{2}$$ (6)

$$Mae\;loss=\left|y_{p}-y_{t}\right|$$ (7)

$$Huber\;loss=\left\{\begin{array}{c} \frac{1}{2}{\left(y_{p}-y_{t}\right)}^{2}\;for\left|y_{p}-y_{t}\right|\leq \delta \\ \delta \left|y_{p}-y_{t}\right|-\frac{1}{2}\delta^{2}\;otherwise \end{array}\right.$$ (8)

$$\mathrm{Log}\cosh loss=\log\left(\cosh\left(y_{p}-y_{t}\right)\right)$$ (9)

where *y*_*p*_ is the predicted value of the group, and *y*_*t*_ is the true value of the group. In Huber loss, the value of δ is determined by cross-validation. In addition to evaluating the model using the mean square error (MSE), we also use the HL statistic and its corresponding *p*-value to test the accuracy and rationality of the predicted value. In the HL test, if the *p*-value is greater than 0.05, it means that the model has passed the HL test, which means that there is no significant difference between the predicted value and the real value, otherwise it means that the model fit is poor. The complete calculation process of the risk based on the SAFE-MIL framework is given in Algorithm 1.

Algorithm 1 SAFE-MIL.

**Input**: *N* training groups{*G*_*1*_, *G*_*2*_,…,*G*_*N*_}.
**Output**: Drug failure risk of *N* groups {*D*_*1*_, *D*_*2*_, … , *D*_*N*_}, Q1, Q2, Q3.
SAFE-MIL (*Epochs, Threshold*)
  Initialize neural network *Net*;
  for (*epoch=1; epoch<=Epochs; epoch++*)
     *GlobalErr*=0; //Set the initial value of global error to be zero
     for (*i=1; i<=N; i++*)
      Compute the output error *E*_*i*_ of group *B*_*i*_ according to Equation 2;
      *GlobalErr = GlobalErr+E*_*i*_;
      The weights in *Net* are modified according to *E*_*i*_ and the
      weight-updated rule of BP algorithm;
     end
     If (*GlobalErr<=Threshold*)
       return *Net*;
     end
  end
  return *Net*;

## 3 Results

### 3.1 Patient characteristics and somatic variation detection

**FIGURE 1:**
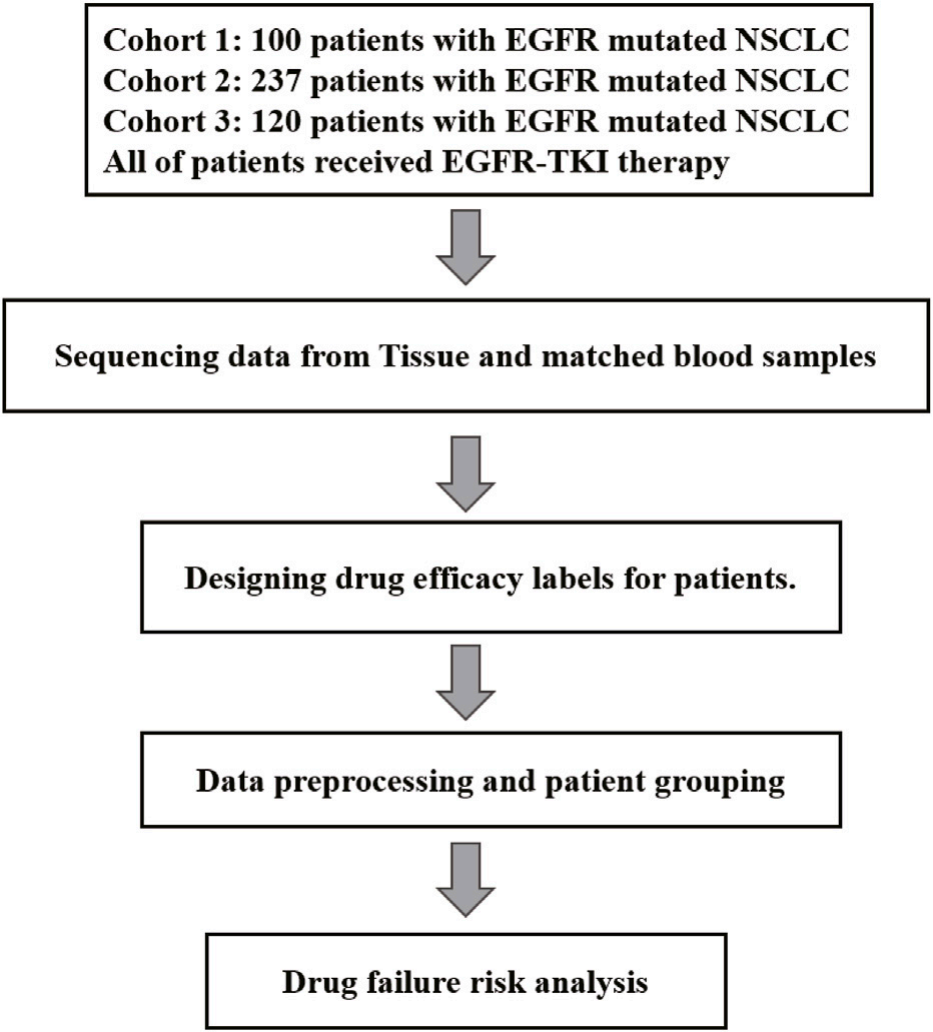
Flow of participants in the study.

Cohort 1 included 100 patients with NSCLC accepted first-line EGFR-TKI target therapy (36% male, 90% stage IV) (Supplementary Table S1). The age at diagnosis ranged from 33 to 80 years, with a median of 61 years (Table 1). In the tumor samples, a total of 592 somatic alterations were detected, including 539 SNVs (single nucleotide variants, SNVs) and small indels (insertion and deletions, indels), 50 copy number variants (CNVs), and 1 structural variants (SVs) (Supplementary Table S2). Cohort 2 comprised 237 patients with NSCLC accepted EGFR-TKI target therapy (40% male, stage IV) (Supplementary Table S3), with ages at diagnosis ranging from 26 to 86 years and a median age of 60 years (Table 1). In the tumor samples, a total of 1738 somatic alterations were detected, including 1,454 SNVs, 268 CNVs, and 4 SVs (Supplementary Table S4). Cohort 3 comprised 120 patients with NSCLC accepted EGFR-TKI target therapy (43.3% male, stage IV) (Supplementary Table S5), with ages at diagnosis ranging from 35 to 83 years (Table 1). In the tumor samples, a total of 930 SNVs or Indels, and 12 structural variants (SVs) were detected (Supplementary Table S6). All patients were found to carry EGFR-sensitive mutations. Cohorts 1 and 2, 3 have recorded slightly different survival information, with the cohort one documenting patients’ progression-free survival (PFS), while the cohort 2/3 records time to treatment failure (TTF). In our study, both PFS and TTF were used as evaluation criteria to assess the predictive performance of our algorithm. These two outcomes are related, they showed a high correlation in NSCLC of targeted therapy, immunotherapy, and chemotherapy. Especially in targeted therapies of EGFRm-TKI, the correlation is even higher (r = 0.91, 95% CI 0.90,0.92) (Blumenthal et al., 2019).While PFS focuses specifically on disease progression, TTF offers a broader perspective by considering various factors influencing treatment continuation. The study utilized clinical data from patients and somatic alterations for the SAFE-MIL investigation. The study workflow is illustrated in Figure 1.

### 3.2 The result of SAFE-MIL for predicting EGFR-TKI failure risk in patients with EGFR-mutated NSCLC

**FIGURE 2:**
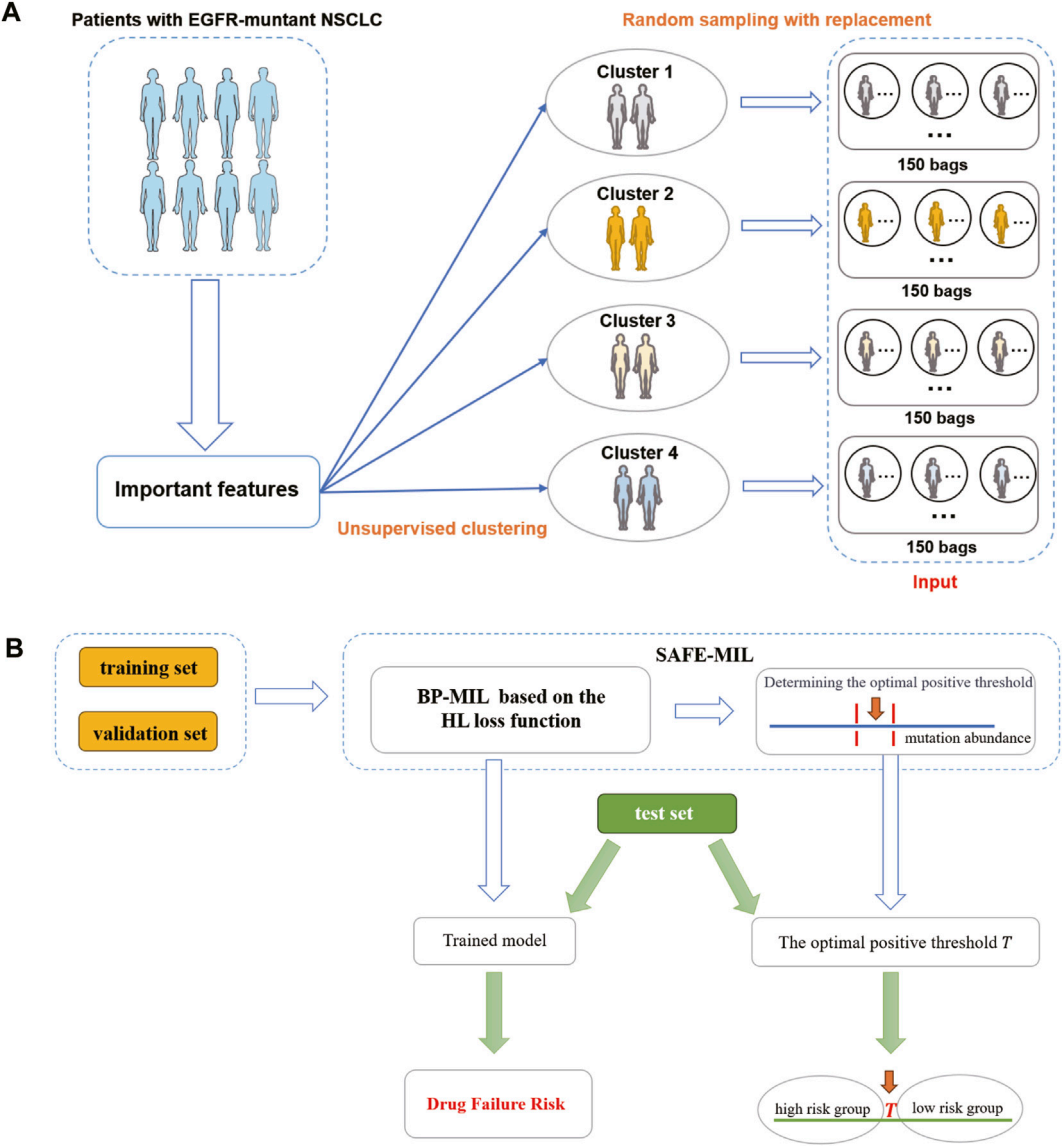
The flowchart of SAFE-MIL. **(A)** Data preprocessing process. First, cluster patients into 4 clusters using unsupervised learning. Then, randomly select 1–10 patients from each cluster to form a group, with each patient serving as an instance. **(B)** SAFE-MIL workflow. SAFE-MIL learns the representation of drug failure risk for each patient group through the HL loss function and determines the optimal positive threshold based on mutation abundance level.

The workflow of this study is shown in Figure 2B. We collected clinical data and SNV mutation data of 457 EGFR-mutated NSCLC patients and conducted data preprocessing. Following the mentioned criteria from the preceding text, we calculated the drug effectiveness label for each patient. Subsequently, an unsupervised clustering of patients was performed using the k-means algorithm based on the features including age, gender, mutation abundance, TP53 co-mutated or not, and the number of co-mutations, which were outlined in Table 3. Consequently, we normalized these features using min-max normalization and employed them for unsupervised clustering using the k-means algorithm. After the feature selection process, mutation abundance was identified as the most significant feature and selected as a key predictor. Although the presence of co-TP53 mutation and the number of co-mutations are two important genomic molecular features, but in this study the two features were indicated not important. The reasons may be from two aspects: firstly, the enrolled population was exclusively EGFR-mutant, with a relatively homogeneous molecular subtype; secondly, the data used in this study originated from a panel sequencing of ∼ 1Mb, which may have limited the representation of the feature regarding the number of co-mutations compared to whole-exome sequencing data. Finally, we randomly selected 1 to 10 patients from each cluster using a with-replacement sampling method to generate groups. The drug effectiveness label of each group was determined by the average effectiveness label of the instances within the group.

**TABLE 3:** The input features of SAFE-MIL.

Feature	Data type	Description
Age	Numerical	The age of patients
Gender	Categorical	The gender of patients. A value of 1 corresponds to female, while 0 corresponds to male
Mutation abundance level	Numerical	Mutation Abundance level/Copy Number
TP53	Categorical	Does the patient have a TP53 gene mutation? A value of 1 indicates presence, while 0 indicates absence
The number of co-mutated genes	Numerical	The number of co-mutated genes

**FIGURE 3:**
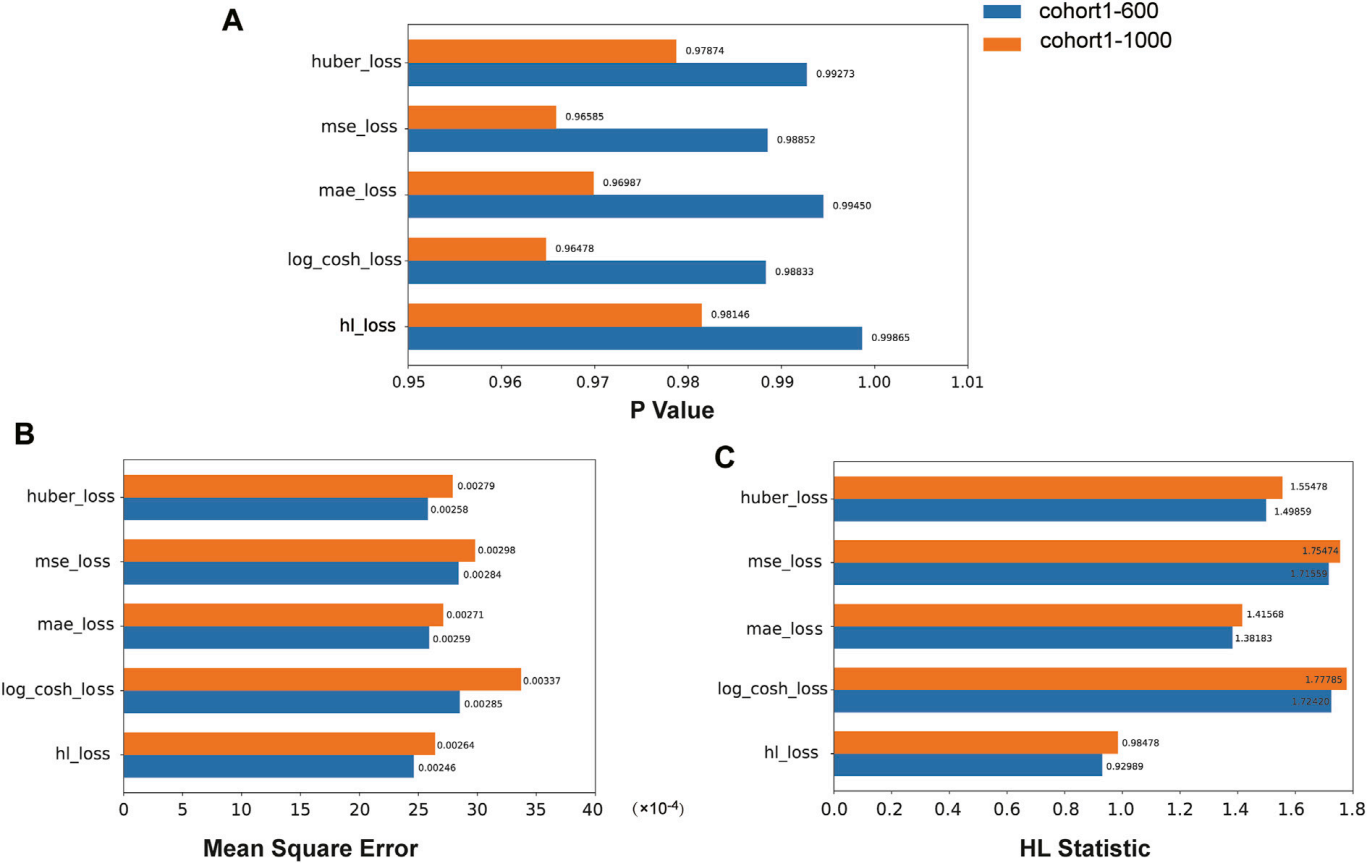
The performance of the HL loss function and four traditional regression loss functions were compared on cohort one in terms of *P*-value **(A)**, mean squared error **(B)**, and HL statistic **(C)**.

**FIGURE 4:**
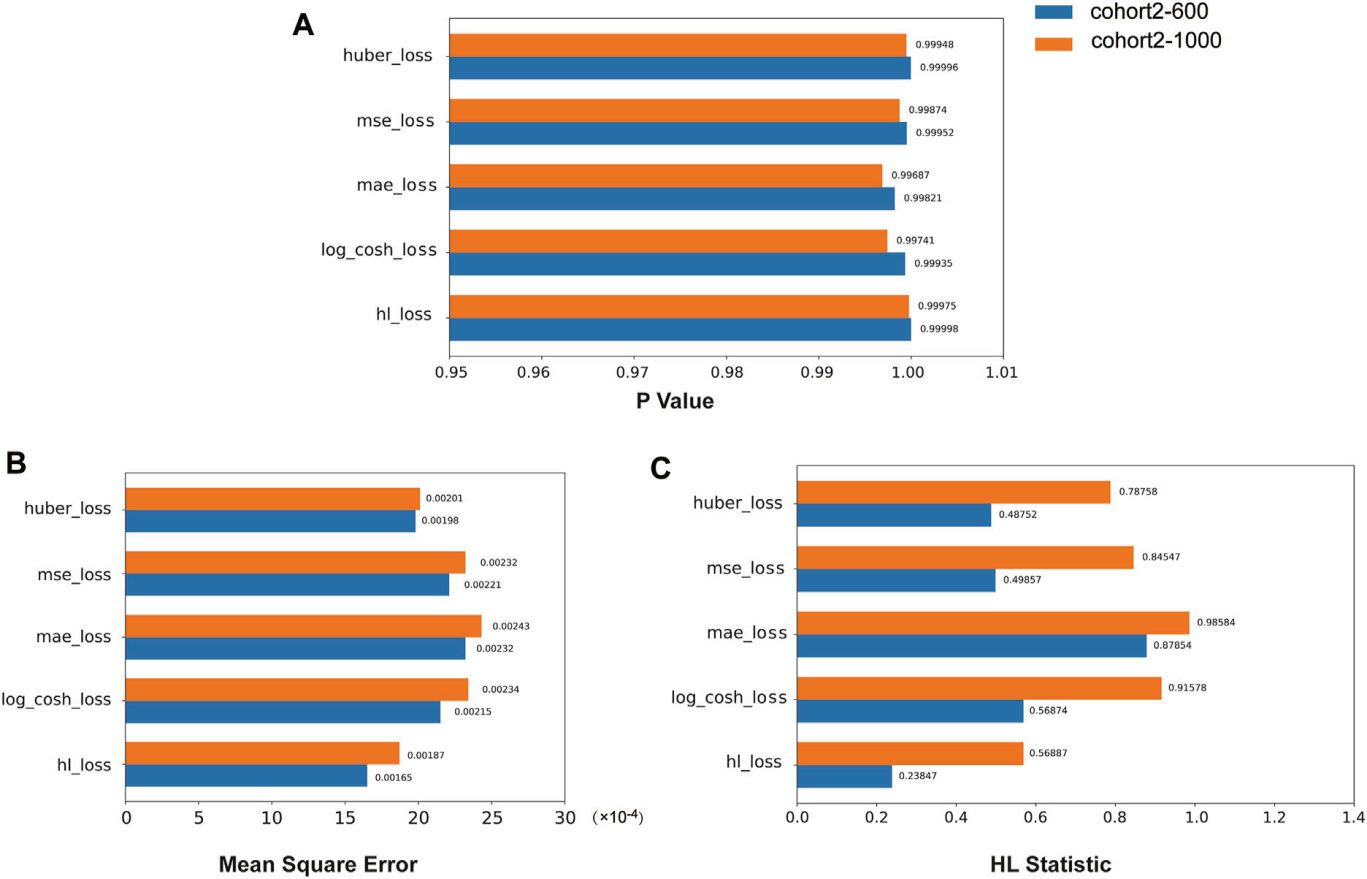
The performance of the HL loss function and four traditional regression loss functions were compared on cohort 2 in terms of *P*-value **(A)**, mean squared error **(B)**, and HL statistic **(C)**.

**FIGURE 5:**
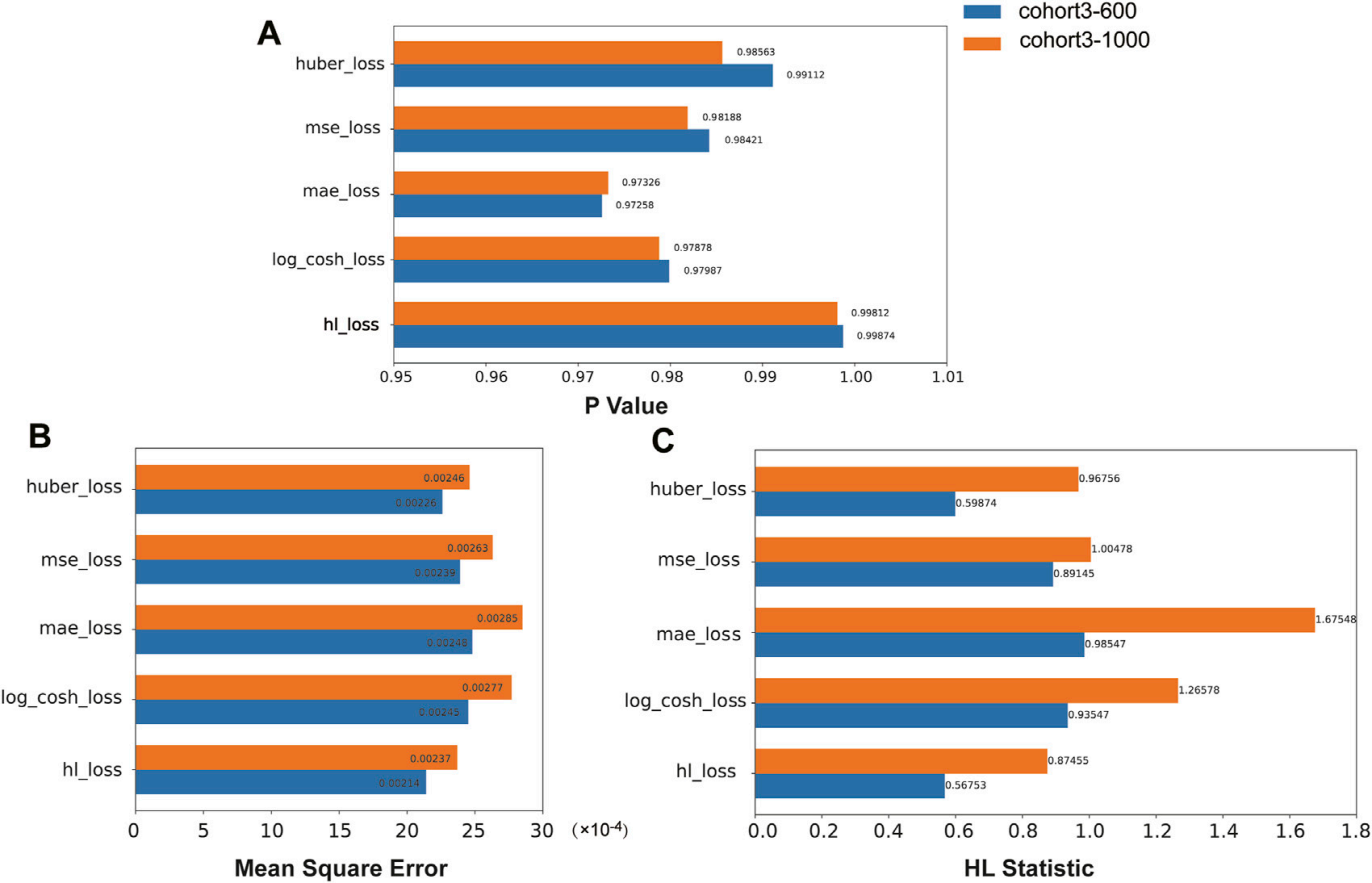
The performance of the HL loss function and four traditional regression loss functions were compared on cohort 3 in terms of *P*-value **(A)**, mean squared error **(B)**, and HL statistic **(C)**.

We compared the performance of the HL loss function and four traditional regression loss functions in terms of mean squared error (MSE), HL statistic, and *P*-value. In the cohort1, overall, all loss functions passed the HL test with a *p*-value greater than 0.05 (Figure 3A), and achieved low mean squared error (MSE) (Figure 3B). SAFE-MIL demonstrated the lowest MSE and HL statistics among the two generated datasets in predicting drug effectiveness (Figures 3B, C), indicating that the HL-based loss functions can capture differences among instances within groups, ensuring accurate estimation results while maintaining statistical interpretability. Similarly, in the cohort2, SAFE-MIL based on the HL loss also achieved lower MSE (Figure 4). Due to the larger sample size of cohort2 compared to cohort1, theoretically, the model of HL-based SAFE-MIL predictive performance should be somewhat better. In summary, HL-based SAFE-MIL achieved the lowest MSE and passed the HL test in both datasets, obtaining optimal HL statistics. Similarly, in the cohort2 and cohort3, SAFE-MIL based on the HL loss also achieved lower MSE (Figures 4, 5). Due to the larger sample size of cohort 2 compared to cohort1, theoretically, the model of HL-based SAFE-MIL predictive performance should be somewhat better. In summary, HL-based SAFE-MIL achieved the lowest MSE and passed the HL test in all three datasets, obtaining optimal HL statistics.

**FIGURE 6:**
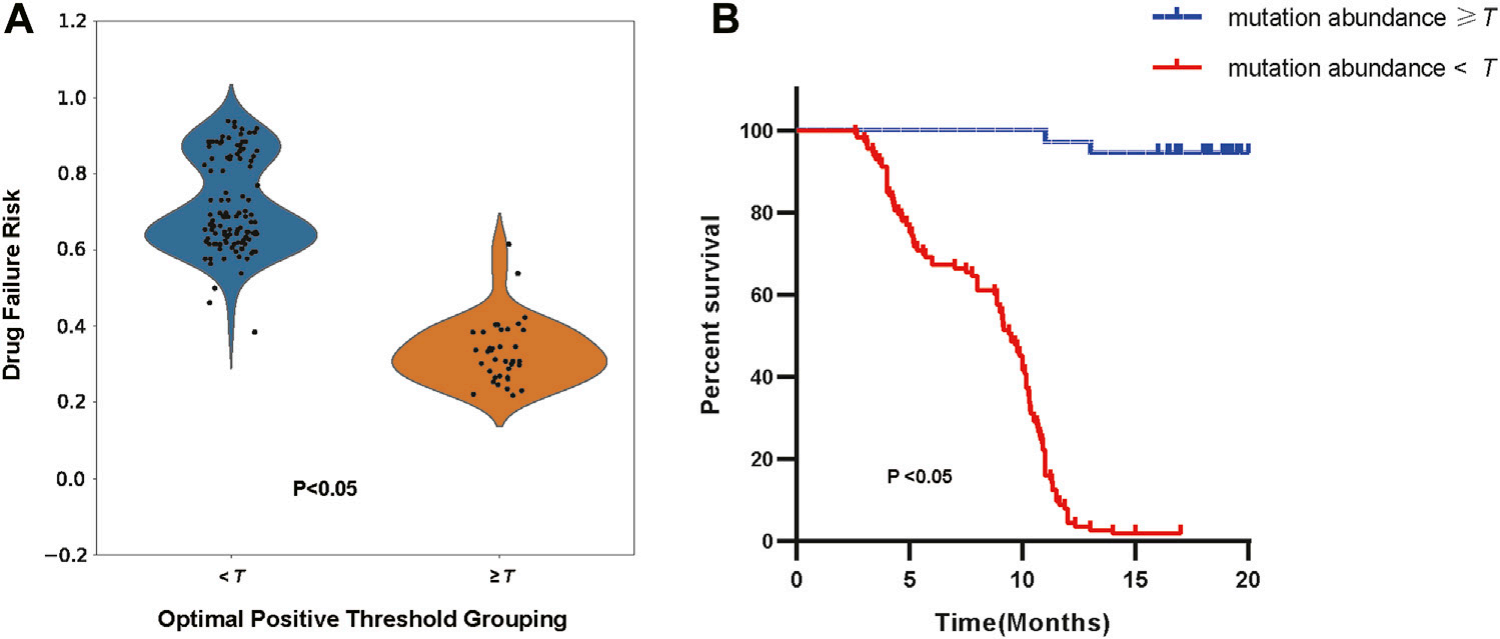
**(A)** The distribution of drug failure risk under the optimal positive threshold grouping (Independent Samples t-test, *p* < 2.2e-16). **(B)** The survival analysis of patients according to the optimal positive threshold (Log-rank test, *p* = 2.8e-9).

Additionally, we selected cohort1-600 as examples to illustrate the relationship between the optimal positive threshold and drug efficacy. Based on the optimal positive threshold determined of mutation abundance, we divided patients in cohort1-600 into two groups to observe differences in drug failure risk and survival outcomes. Following the calculation rules mentioned earlier, the optimal positive threshold for mutation abundance in cohort1-600 was determined to be τ = 0.479. Firstly, using the threshold τ, we categorized patients into high-risk (mutation abundance level < τ) and low-risk (mutation abundance level >= τ) groups. In cohort1-600, the average drug failure risk in the high-risk group was 0.715, significantly higher than the 0.332 in the low-risk group (t-test, *p*< 2.2e-16, Figure 6A). Since survival information for patients in cohort1-600 was recorded, we evaluated the survival differences between the high-risk and low-risk groups in cohort1-600. The results indicated that the average progression-free survival in the high-risk group in cohort1-600 was 8.4 months, significantly lower than the 16.4 months in the low-risk group (Log-rank test, *p* = 2.8e-9, Figure 6B).

The optimal positive threshold identified by SAFE-MIL represents the mutation abundance level at which patients are stratified into high-risk and low-risk groups with respect to treatment failure. This threshold is determined based on statistical analysis and represents a critical point for distinguishing patients who are more likely to experience treatment failure from those who are likely to respond favorably to therapy. Patients identified as high-risk above the threshold may benefit from closer monitoring, alternative treatment options, or enrollment in clinical trials for novel therapies. Conversely, patients below the threshold may be considered lower risk and may continue with standard therapy with confidence in treatment efficacy. The use of the optimal positive threshold enhances patient risk stratification by providing a quantitative measure that correlates with treatment outcomes. This allows for more accurate risk assessment and personalized treatment approaches, ultimately improving patient outcomes and optimizing resource allocation in healthcare settings. The identification of an optimal positive threshold enables clinicians to make informed decisions regarding treatment selection by considering individual patient risk profiles. SAFE-MIL facilitates precision medicine approaches, ensuring that patients receive the most appropriate and effective treatments based on their unique characteristics.

## 4 Discussion

The traditional clinical decision-making approaches often simplify drug efficacy into binary classifications, which fails to capture the nuanced risks associated with drug failure in individual patients. To address this limitation, we proposed that the clinical drug response for patients should be a regression value, reflecting individual variability in drug failure risk. This study introduces the SAFE-MIL framework, a new method for calculating and predicting the risk of drug failure in patients, which designs drug effectiveness labels based on clinical features of patients and builds a model to predict the risk of drug failure using multiple instance learning. A critical enhancement in SAFE-MIL is the integration of a loss function derived from the Hosmer-Lemeshow test. This integration not only boosts model performance but also ensures statistical interpretability, enabling robust assessments of drug failure risks and facilitating patient stratification by identifying the optimal positive threshold. Our results indicate that SAFE-MIL outperforms traditional regression methods in accuracy, particularly in capturing inter-patient variability in risk assessments, and successfully passes the HL test.

The concept of drug failure risk introduced in this study provides a more comprehensive characterization of drug effectiveness. Due to the significant molecular heterogeneity observed in tumors, there are often many different molecular features and feature combinations that can lead to the model predicting specific drug responses. However, elucidating these features and discerning whether they are distinct or functionally linked can be challenging. This is primarily due to the “black box” nature of most machine learning models, which prioritize predictive accuracy over an understanding of the underlying biological mechanisms (Ching et al., 2018). SAFE-MIL addresses this by enhancing clinical interpretability through its novel use of MIL and HL test integration, thereby somewhat bridging the gap between clinical needs and machine learning methodologies.

Furthermore, the study critically evaluates and expands upon existing methods for interpreting deep learning algorithms in clinical settings [(Zhang et al., 2018)]. Our approach incorporates the Hosmer-Lemeshow test into the MIL framework to estimate the risk of treatment failure, significantly improving the model’s predictive calibration performance and offering a unique risk assessment perspective beyond traditional interpretation methods. The results demonstrate that our framework outperforms traditional regression loss functions in terms of accuracy and successfully passes the Hosmer-Lemeshow test, highlighting its capability to accurately capture inter-patient variability in risk while ensuring statistical interpretability. Additionally, our framework enables the identification of an optimal threshold for mutation abundance level, allowing for effective patient stratification into high-risk and low-risk groups. This stratification reveals significant disparities in drug failure risk and progression-free survival, showcasing the utility of our approach in guiding treatment decisions.

Although our study primarily focuses on patients with EGFR-mutant non-small cell lung cancer treated with EGFR tyrosine kinase inhibitors, the potential of SAFE-MIL extends far beyond this specific context. Moving forward, efforts to validate and refine the SAFE-MIL framework across different cancer types and therapeutic modalities will further enhance its utility and broaden its impact on clinical practice. Beyond its predictive capabilities, SAFE-MIL offers early risk identification and optimizing clinical trial design. For instance, SAFE-MIL enables the identification of patient subgroups with distinct risk profiles, paving the way for targeted interventions and precision medicine approaches. Moreover, integrating automated risk assessment tools into clinical workflows holds promise for streamlining decision-making processes and improving patient outcomes in real-world practice. Consequently, the integration of SAFE-MIL is poised to transform clinical practice by boosting treatment efficacy and ensuring patient safety. The drug failure risk metrics derived from SAFE-MIL appear to correlate with the patient’s resistance mechanisms, a relationship that warrants deeper investigation.

Despite these promising results, our study acknowledges several limitations. Firstly, due to the limited number of patients, we could only generate groups through bootstrap sampling, and we constrained the number of groups and the number of instances per group. Additionally, the availability and quality of data sources pose significant challenges to model development and validation. Future efforts should focus on employing SAFE-MIL in larger-scale studies to explore more robust and efficient risk calculation models. Furthermore, implementing SAFE-MIL in real-world healthcare settings necessitates careful consideration of practical challenges such as workflow integration, clinician training, and patient acceptance.

## 5 Conclusion

This paper presented SAFE-MIL, a novel risk assessment framework tailored to address the issue of varying survival benefits among patients with certain target gene mutations undergoing targeted therapy. By incorporating multiple instance learning (MIL), SAFE-MIL constructs patient-specific effectiveness labels and designs a novel interpretable loss function based on the Hosmer-Lemeshow test. This risk assessment framework accurately estimates the risk of treatment failure and also provides the optimal threshold for risk stratification. A comprehensive case study involving 457 non-small cell lung cancer patients with EGFR mutations treated with EGFR tyrosine kinase inhibitors demonstrated that SAFE-MIL outperforms conventional regression methods in accuracy, effectively capturing inter-patient variability in risk. This computational framework possesses statistical interpretability and adaptability, rendering it a helpful tool in clinical decision-making for targeted therapy. It has the potential to advance personalized medicine by enhancing patient stratification methods.

## Data Availability

The original contributions presented in the study are included in the article/Supplementary Material, and the data presented in the study are deposited in the GSA- human repository, accession number HRA006927.
