# Supplementary material for: SAFE-MIL: a statistically interpretable framework for screening potential targeted therapy patients based on risk estimation
Source: Front Genet. 2024 Aug 15;15:1381851. doi: 10.3389/fgene.2024.1381851 (PMC11357964; doi:10.3389/fgene.2024.1381851)
Supplement: Supplementary file 1 [file DataSheet2.PDF]

Supplementary Material

1. Feature selection process

During data preprocessing, we first manually assessed the importance of the features and then used LASSO regression for feature selection (Supplementary Figure 1A) to enable the machine learning model to better understand and process the data, thereby improving model performance and predictive ability. As the log(λ) value increases, some feature coefficients become zero, indicating that these features are not important. When the log(λ) value is around -4, the Mean Squared Error (MSE) of the features reaches its minimum value of 0.018 (Supplementary Figure 1B). A total of four features were retained. Additionally, we manually added the feature "The number of co-mutated genes," resulting in the feature set shown in Table 3. We used these five features for unsupervised clustering.

2. Unsupervised clustering

In multi-instance learning, samples with similar feature values are expected to have similar labels. Large differences in instance labels within a bag can hinder the model's accurate prediction. Additionally, in medicine, different types of patients typically require different treatments to achieve the best therapeutic outcomes. This personalized treatment approach aims to provide the most appropriate therapy based on the specific characteristics and disease conditions of the patients. Therefore, before performing multi-instance learning bagging, we conducted unsupervised clustering on the samples.

When performing unsupervised clustering, we plotted the relationship between the Sum of Squared Errors (SSE) and the number of clusters to determine the optimal number of clusters (Supplementary Figure 1C). The calculation formula for SSE is define as

$$SSE = \sum_{i=1}^{k} \sum_{p=C_i} |p - m_i|^2$$

where C_i is the i-th cluster, p is the sample in C_i, mi is the centroid of C_i (the mean of all samples in C_i), and SSE represents the clustering error of all samples, indicating the quality of the clustering. As shown in Supplementary Figure 1C, SSE gradually decreases as the number of clusters k increases, and the magnitude of the decrease in SSE determines the degree of sample aggregation. When k is equal to 4, the magnitude of the decrease in SSE is the largest, indicating that k=4 is the optimal number of clusters. Supplementary Figure 1D shows the scatter diagram of all samples compressed into two-dimensional space. Finally, we randomly selected 1 to 10 patients from each cluster using a with-replacement sampling method to generate groups. The drug effectiveness label of each group was determined by the average effectiveness label of the instances within the group.

3．The p-value of Hosmer-Lemeshow

The Hosmer-Lemeshow test is a statistical method used to evaluate the goodness-of-fit for binary

logistic regression models. The basic idea is to divide the data into several groups and then compare the actual observed values with the predicted values in each group. In the Hosmer-Lemeshow test, the p-value is calculated based on the chi-squared distribution, corresponding to the Hosmer-Lemeshow statistic's cumulative distribution function value （Equations (2) and (3) in the manuscript.） . If the p-value is very small (less than 0.05), it indicates that the model does not fit well, meaning there is a significant difference between the predicted probabilities and the actual observed values. If the p-value is large, it indicates that the model fits well, meaning there is no significant difference between the predicted probabilities and the actual observed values.

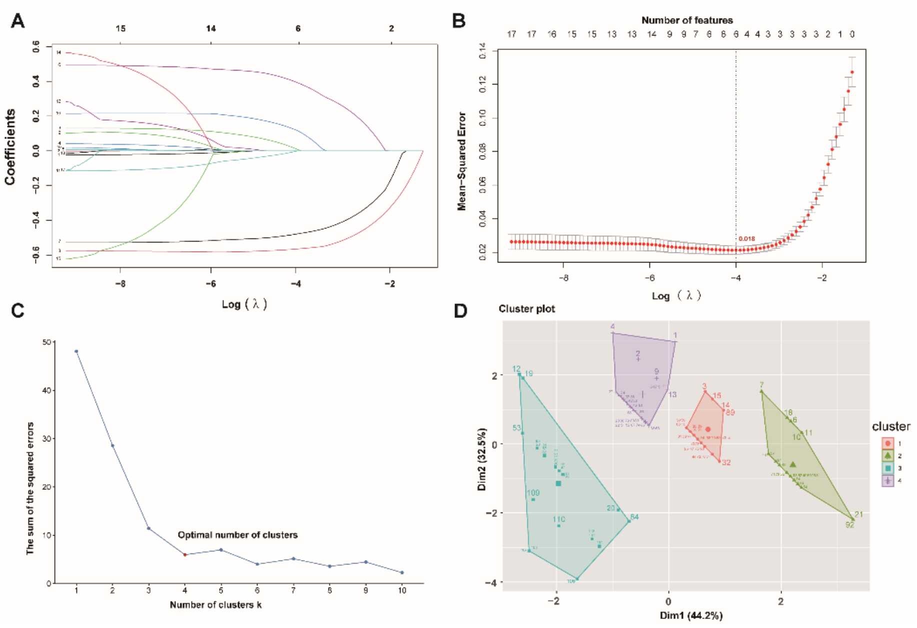

Supplementary Figure 1. (A) The change curve of the coefficient of the features with the increase of the log (λ) value. (B) The relationship between the mean squared error of the feature and the log (λ) value. (C) The relationship between the number of clusters and sum of the squared errors in unsupervised clustering. (D) The scatter diagram of all samples compressed into two-dimensional space.
